# Supplementary material for: Integrated Diabetes Self-Management (IDSM) mobile application to improve self-management and glycemic control among patients with Type 2 Diabetes Mellitus (T2DM) in Indonesia: A mixed methods study protocol
Source: PLoS One. 2022 Nov 28;17(11):e0277127. doi: 10.1371/journal.pone.0277127 (PMC9704669; doi:10.1371/journal.pone.0277127)
Supplement: S3 File — (PDF) [file pone.0277127.s003.pdf]

FOCUS GROUP DISCUSSION GUIDELINES

For Nurses in Public Health Center

Group discussion :
Date :
Time :
Places :
Moderator :
Notulen :
Observer :
Name and participant code

1. …………………… participant code : P1
2. …………………… participant code : P2
3. …………………… participant code : P3
4. …………………… participant code : P4
5. …………………… participant code : P5
6. …………………… participant code : P6
7. …………………… participant code : P7
8. …………………… participant code : P8
9. …………………… participant code : P9
10. …………………. participant code : P10

A. PREPARATION
   1. Onsite Meeting
      a. Participants wash their hands with soap / hand sanitizer that has been provided by the researcher
      b. Participants will be given a mask by the researcher
      c. Participants enter the room and occupy the seats that have been provided and are arranged at a distance by the researcher
      d. The moderator will start the discussion by opening
   2. Online Meeting
      a. Make sure that the device is connected to the internet
      b. Download the meeting app via play store/app store
      c. Open the meeting app link that has been given by the researcher

d. Rename the account name with the original name format
e. Participants dress politely and neatly and activate video cameras during the discussion
f. The moderator will start the discussion by opening

C. OPENING

1. Greeting
2. Introducing
3. Opening the event by praying
4. Conveying the purpose of the discussion

D. CONVEYING PROCEDURES

1. FGD will take place for 60-90 minutes
2. The discussion will be guided by the researcher as a moderator and assisted by research assistants as observers and note takers
3. Moderator will ask questions and participants respond
4. All members of the group have the same right to participate and express their opinions by raising their hands first before answering
5. Participants' answers are free and do not have to be the same as other participants
6. All opinions are right and nothing is wrong
7. Participants' opinions are very important and very meaningful in this study
8. Participants can leave the discussion by asking the moderator for permission
9. All answers will be recorded and names withheld on FGD reports using participant code.
10. The observer will observe the course of the FGD, and helps the moderator monitor the timing, focus of the FGD and participants
11. The note taker will note the core issues discussed and group dynamics

E. IMPLEMENTATION

1. All participants introduced themselves
2. Moderator asks questions :
   1) What do you think about diabetes self-management?
   2) What should be regulated in diabetes self-management?
   3) How to apply diabetes self-management in providing health services to diabetic patients?
   4) How do you monitor self-management of diabetic patients?
   5) How to monitor self-management of diabetic patients can be done properly?

6) What guidelines do you use in monitoring the implementation of self-management of diabetic patients?
7) How do you help diabetic patients overcome problems in self-management of diabetes?
8) What do you think if diabetes self-management is carried out in an integrated between patients, families and nurses?
9) What do you think about the role of patients, families and nurses in the integrated diabetes self-management?
10) What are the difficulties that will be found in implementing integrated diabetes self-management (patient, family and nurse)?
11) What do you think if the implementation of integrated diabetes self-management uses clear guidelines?
12) What do you think if the implementation of diabetes self-management is integrated using a guide in the form of an Android application on a Smartphone?
13) What should be done so that users can operate an Android-based integrated diabetes self-management application?
14) What kind of Android-based integrated diabetes self-management application do you want?
15) What features should be included in an Android-based integrated diabetes self-management application?

F. CLOSING

1. Conclude the results of the group discussion
2. Say thank you to the members of the FGD
3. Closing the FGD by praying together
