# Supplementary material for: Integrated Diabetes Self-Management (IDSM) mobile application to improve self-management and glycemic control among patients with Type 2 Diabetes Mellitus (T2DM) in Indonesia: A mixed methods study protocol
Source: PLoS One. 2022 Nov 28;17(11):e0277127. doi: 10.1371/journal.pone.0277127 (PMC9704669; doi:10.1371/journal.pone.0277127)
Supplement: S5 File — (PDF) [file pone.0277127.s005.pdf]

INTERVIEW GUIDE FOR THE STAFFS IN CHARGE OF BPJS HEALTH

Characteristics of participants

Participant's initial name :
Age :
Gender :
Education :
Marital status :
Long time on duty :

A. PREPARATION
1. Onsite Interview
   a. Interviewer and participant wash their hands with soap / hand sanitizer
   b. Interviewer and participant use a mask
   c. The interviewer and the participant sit opposite each other and are spaced apart
   d. The interviewer will start the interview by opening
2. Online Interview
   a. Make sure that the device is connected to the internet
   b. Download the meeting app via play store/app store
   c. Open the meeting app link that has been given by the researcher
   d. Rename the account name with the original name format
   e. Participants dress politely and neatly and activate video cameras during the discussion
   f. The interviewer will start the interview by opening

C. OPENING
1. Greeting
2. Introducing
3. Conveying the purpose of the interview

D. IMPLEMENTATION
1. Interviewer asks questions :

Question	Probes	Note
What is your perception about the current development of PROLANIS activities, especially for Type 2 Diabetes Mellitus patients?	- Reason<br>- Purpose	
How do you apply self-management to diabetic patients in providing health services?	-	
How do you monitor self-management of diabetic patients?	- diet<br>- exercise<br>- medication<br>- self-monitoring of blood sugar<br>- examination and foot care<br>- stress management	
How to monitor self-management of diabetic patients can be done properly?	- Barrier<br>- Support	
What guidelines do you use in monitoring the implementation of self-management of diabetic patients?	- Book<br>- Android App	
How do you help diabetic patients overcome problems in self-management of diabetes?	-	
What do you think if diabetes self-management is carried out in an integrated between patients, families and nurses?	- Barriers<br>- Support	
What do you think about the role of each element in the integrated diabetes self-management?	- Role of patient<br>- Role of family<br>- Role of nurse	
What do you think if the implementation of diabetes self-management is integrated using a guide in the form of an android application on a smartphone?	-	
What should be done so that users can operate an Android-based integrated diabetes self-management application?	- Provide socialization<br>- Provide training<br>- Provide modul	
What kind of Android-based integrated diabetes self-management application do you want?	- Operating system<br>- Features<br>- Smartphone app display	
How does BPJS Kesehatan cover insurance for PROLANIS members, especially Diabetes patients?	- Medical examination<br>- Treatment	
what do you think about rewards and punishments for PROLANIS participants who carry out PROLANIS activities and self-management activities?	- Reward<br>- Punishment	

E. CLOSING

1. Say thank you to the partisipan
2. Say that later if there is still data needed, we will meet again with the participants (end time contract)
