# Supplementary material for: Integrated Diabetes Self-Management (IDSM) mobile application to improve self-management and glycemic control among patients with Type 2 Diabetes Mellitus (T2DM) in Indonesia: A mixed methods study protocol
Source: PLoS One. 2022 Nov 28;17(11):e0277127. doi: 10.1371/journal.pone.0277127 (PMC9704669; doi:10.1371/journal.pone.0277127)
Supplement: S6 File — (PDF) [file pone.0277127.s006.pdf]

HEURISTIC EVALUATION GUIDELINE

A. PRELIMINARY

1. Participants will be given an explanation and informed consent
2. Researcher introduces herself and conveying the purpose of the meeting

B. IMPLEMENTATING

1. In the first round:
   a. Three Experts as evaluators are given software an Android-based IDSM Application, usage modules and HE checklists.
   b. Experts will be asked to operate the Android-based IDSM Application and conduct independent evaluations by writing down the problems found in accordance with HE principles. Each violation is noted as a usability problem.
2. In the second round:
   a. The evaluators discussed the problems that had been identified and combined in a meeting convened by the evaluators and were given a list of problems.
   b. A problem list is the same problem that has been identified by three evaluator and agreed upon among all evaluators.
   c. Any disagreements regarding the problems identified and assigning them to specific heuristics are resolved by consensus.

HEURISTIC EVALUATION CHECKLIST

Heuristic #1: Visibility of system status.

#	Review Checklist	Yes	No	N/A	Comments
1.1	Is there some form of system feedback for each operator action?	☐	☐	☐	
1.2	If pop-up windows are used to display error messages, do they allow the user to view the error field?	☐	☐	☐	
1.3	In a multi-page data entry screen, is each page labeled to indicate its relationship to other pages?	☐	☐	☐	
1.4	Is high informative content placed in a high hierarchical area?	☐	☐	☐	
1.5	Do all the items in the list on the same page? Are they sorted in an order that fits the needs of the task?	☐	☐	☐	
1.6	If a list of items can be sorted by different criteria, does it provide an option to sort them using those criteria?	☐	☐	☐	
1.7	If the list contains items that fall into different categories, is there a filter for the user to narrow down the number of elements they need to check?	☐	☐	☐	
1.8	If the list contains only one item, is the user directly redirected to that item?	☐	☐	☐	
1.9	If the list contains slowly downloading items (e.g., images), is the list divided into multiple pages to display only one page at a time?	☐	☐	☐	
1.10	For articles spanning multiple pages, is the pagination shown at the bottom? Are there links to each individual page, not just the previous and next pages?	☐	☐	☐	
1.11	Is the logo meaningful, identifiable, and sufficiently visible?	☐	☐	☐	
1.12	Are there links to detailed information about the company, website, webmaster, etc.?	☐	☐	☐	
1.13	Are there ways of contacting with the enterprise?	☐	☐	☐	

1.14	In articles, news, reports, etc. Is the author, source, date and review information clearly displayed?	☐	☐	☐	
1.15	For physical location information on the website, are links to maps provided and directions clearly accessible?	☐	☐	☐	
1.16	Is the response time appropriate for the user's cognitive processing?	☐	☐	☐	
1.17	Are response times appropriate for the task?	☐	☐	☐	
1.18	If there is an observable delay (more than fifteen seconds) in system response time, is the user kept informed of the system's progress?	☐	☐	☐	
1.19	Is latency sufficiently reduced?	☐	☐	☐	
1.20	Are splash screens too long avoided?	☐	☐	☐	
1.21	If the download time is more than 20 seconds, is a progress bar offered instead of an uninformative download screen?	☐	☐	☐	
1.22	Is there any visual feedback in the menu or dialog box about which option to choose?	☐	☐	☐	
1.23	Is the current status of an icon clearly indicated?	☐	☐	☐	
1.24	Is there visual feedback when objects are selected or moved?	☐	☐	☐	
1.25	Is the link recognizable? Are there characterizations according to circumstances (visiting, active, etc.)?	☐	☐	☐	
1.26	Are low discoverable areas as touch buttons well identifiable?	☐	☐	☐	
1.27	When swiping gestures are possible, are visible hints offered to the user? Is swiping used with a unique meaning on the same screen?	☐	☐	☐	
1.28	Are expandable menus used sparingly? Do menu labels clearly indicate that they extend to a set of options?	☐	☐	☐	
1.29	When users access the site from a mobile, are they redirected to the mobile version of the site?	☐	☐	☐	
1.30	Is a link to the mobile site provided on the desktop version of the site?	☐	☐	☐	

1.31	Is a link to the full site included on the mobile page?	☐	☐	☐	
1.32	For interface design on mobile, Is clear and concise information about system status provided on each screen?	☐	☐	☐	
1.33	Is important and contextual information, such as battery status, network status, environmental conditions, etc. prioritized?	☐	☐	☐	
1.34	Is sound and light feedback explored as an alternative to text feedback? However, these resources should be used wisely to avoid disturbing users.	☐	☐	☐	
1.35	Does the device keep users informed of all processes and status changes via comments within a reasonable timeframe?	☐	☐	☐	

Heuristic #2: User control and freedom

#	Review Checklist	Yes	No	N/A	Comments
2.1	Users often select system functions by mistake, is there an option marked with "emergency exit" for the user to leave the unwanted state without having to go through an extended dialog?	☐	☐	☐	
2.2	Are the undo and redo actions performed by the user supported by the system?	☐	☐	☐	
2.3	Can users move forward and backward between fields or dialogue box options?	☐	☐	☐	
2.4	If the system has a multi-page data entry screen, can the user move back and forth between all the pages in the batch?	☐	☐	☐	
2.5	If the system uses a question and answer interface, can the user return to the previous question or skip to the next question?	☐	☐	☐	
2.6	Are exits clearly marked?	☐	☐	☐	
2.7	Is the general web site structure user-oriented?	☐	☐	☐	
2.8	Is there a way to let the user know where they are and how to cancel their navigation?	☐	☐	☐	

2.9	Is accidental activation avoided or foreseen (a back button is offered)?	☐	☐	☐	
2.10	In mobile websites, is navigation on the homepage provided?	☐	☐	☐	
2.11	Can users set their own system, session, file, and screen defaults?	☐	☐	☐	
2.12	When a user task is finished, does the system wait for a signal from the user before processing?	☐	☐	☐	
2.13	Is the user being asked to confirm a command that has drastic and damaging consequences?	☐	☐	☐	
2.14	Can users easily reverse their actions?	☐	☐	☐	
2.15	Can users cancel out of operations in progress?	☐	☐	☐	
2.16	If the system has multiple menu levels, is there a mechanism that allows the user to return to the previous menu?	☐	☐	☐	
2.17	Is the menu broad (many items on the menu) rather than deep (many levels of the menu)?	☐	☐	☐	
2.18	If the user can return to the previous menu, can they change the previous menu selection?	☐	☐	☐	
2.19	Are interruptions made by the user treated naturally by the system?	☐	☐	☐	
2.20	Is the user given the possibility to immediately restart his actions after an interrupt?	☐	☐	☐	
2.21	Are basic navigation controls available on the screen even though the device itself provides buttons to perform this function?	☐	☐	☐	
2.22	Should the horizontal and/or vertical navigation options offered by the system be intuitive?	☐	☐	☐	
2.23	Are these options (undo, redo, exits) available via physical buttons or equivalent?	☐	☐	☐	

Heuristic #3: Match between system and real world

#	Review Checklist	Yes	No	N/A	Comments

3.1	Does the system speak the user's language, with words, phrases and concepts that are familiar to the user, rather than system-oriented terms?	☐ ☐ ☐	
3.2	Is real-world convention followed in the system?	☐ ☐ ☐	
3.3	Is information displayed in a logical and natural order?	☐ ☐ ☐	
3.4	Are metaphors properly used as visual clues?	☐ ☐ ☐	
3.5	Are icons concrete and familiar?	☐ ☐ ☐	
3.6	If the shape is used as a visual cue, does it fit with cultural conventions?	☐ ☐ ☐	
3.7	Does the color chosen match the general expectations about color coding?	☐ ☐ ☐	
3.8	If the site uses a hierarchical structure, are the depth and height balanced?	☐ ☐ ☐	
3.9	Is a navigation map or table of contents included on the site?	☐ ☐ ☐	
3.10	Is too much navigation avoided?	☐ ☐ ☐	
3.11	Are menu options sorted in the most logical way, given the user, item name, and assignment variable?	☐ ☐ ☐	
3.12	Do menu choices fit logically into categories that have easy-to-understand meaning?	☐ ☐ ☐	
3.13	Are menu titles parallel grammatically?	☐ ☐ ☐	
3.14	In the navigation menu, are the number of items and terms per item controlled to avoid memory overload?	☐ ☐ ☐	
3.15	Do related and interdependent fields appear on the same screen?	☐ ☐ ☐	
3.16	For the question and answer interface, are the questions stated in clear and simple language?	☐ ☐ ☐	
3.17	Is the language used the same target users speak?	☐ ☐ ☐	
3.18	Is the language clear and concise?	☐ ☐ ☐	
3.19	Does the site follow the rule "1 paragraph=1 idea"?	☐ ☐ ☐	
3.20	Does the system automatically enter a leading or trailing space to align the decimal point?	☐ ☐ ☐	

3.21	Does the system automatically enter dollar signs and decimals for monetary entries?	☐	☐	☐	
3.22	Does the system automatically enter commas in numeric values greater than 9999?	☐	☐	☐	
3.23	What are right-aligned integers and decimal-aligned real numbers?	☐	☐	☐	
3.24	The most natural and easy-to-use mode of operation is obtained by making the same experience of daily life events, is the natural mode extended to smartphones?	☐	☐	☐	
3.25	Do movements such as sliding, dragging, twisting that are applied in everyday life seem/look natural?	☐	☐	☐	
3.26	Does the app rely on user models derived from previous technologies?	☐	☐	☐	

Heuristic #4: Consistency and standards

#	Review Checklist	Yes	No	N/A	Comments
4.1	Does the app make users wonder if different words, situations, or actions mean the same thing?	☐	☐	☐	
4.2	Are platform conventions followed?	☐	☐	☐	
4.3	Is limiting orientation avoided? (Users tend to switch orientations when deadlocks occur and, if the app doesn't support them, their flow will be interrupted, and they'll wonder why it's not working)	☐	☐	☐	
4.4	Is the navigation (horizontal and vertical) consistent across orientations? (Some apps use different navigation directions in the two orientations; for example, they use horizontal navigation in landscape and vertical navigation in portrait).	☐	☐	☐	
4.5	Is content consistent across orientations?	☐	☐	☐	
4.6	Are attention-getting techniques used with care?	☐	☐	☐	

4.7	Is intensity maintained in two levels only?	☐ ☐ ☐	
4.8	Is the number of colors used limited to four? Are additional colors saved for occasional use only?	☐ ☐ ☐	
4.9	Are the colors far apart along the visible spectrum?	☐ ☐ ☐	
4.10	Are soft tones used for regular positive feedback and loud for rare critical conditions?	☐ ☐ ☐	
4.11	If the system has a multi-page data entry screen, do all the pages have the same title?	☐ ☐ ☐	
4.12	Do online instructions appear in consistent locations across screens?	☐ ☐ ☐	
4.13	Have industry or company standards been established for menu design, and are these standards consistently applied to all menu screens in the system?	☐ ☐ ☐	
4.14	Are there no more than twelve to twenty icon types?	☐ ☐ ☐	
4.15	Has a heavy use of all uppercase letters on a screen been avoided?	☐ ☐ ☐	
4.16	Is there a consistent icon design scheme and style maintenance across the system?	☐ ☐ ☐	
4.17	Are menu choice lists presented vertically?	☐ ☐ ☐	
4.18	If "exit" is a menu option, does it always appear at the bottom of the list?	☐ ☐ ☐	
4.19	Are menu titles either centered or left-justified?	☐ ☐ ☐	
4.20	Are field labels consistent from one data entry screen to another?	☐ ☐ ☐	
4.21	Do field labels appear to the left of single fields and list fields above?	☐ ☐ ☐	
4.22	Are field and field labels typographically differentiated?	☐ ☐ ☐	
4.23	Is the data entry value structure consistent from screen to screen?	☐ ☐ ☐	
4.24	Are system objects named consistently across all commands in the system?	☐ ☐ ☐	

4.25	Are user actions named consistently across all commands in the system?	☐	☐	☐	
4.26	Are menu selection names consistent, both within each menu and across systems, in grammatical style and terminology?	☐	☐	☐	
4.27	Does the menu selection name structure match the appropriate menu title?	☐	☐	☐	
4.28	Does the menu structure match the task structure?	☐	☐	☐	
4.29	When the prompt implies a required action, are the words in the message consistent with that action?	☐	☐	☐	
4.30	Where is the website going? Are they well defined? Is the content and services provided fit for this purpose?	☐	☐	☐	
4.31	Is the look & feel appropriate for the purpose, characteristics, content and services of the website?	☐	☐	☐	
4.32	Is the web site updated frequently?	☐	☐	☐	
4.33	Is system response after clicking links predictable?	☐	☐	☐	
4.34	Are nowhere links avoided?	☐	☐	☐	
4.35	Are orphan pages avoided?	☐	☐	☐	
4.36	Is the mobile interface similar to the desktop interface? In terms of buttons, logos and color schemes for a consistent user experience across platforms?	☐	☐	☐	
4.37	Are link labels emphasized in graphic elements?	☐	☐	☐	
4.38	Does the app maintain components in the same place and view all interactions?	☐	☐	☐	
4.39	Are similar functions performed by similar interactions?	☐	☐	☐	
4.40	Is the metaphor of each component or feature unique across the application, to avoid misunderstandings?	☐	☐	☐	

Heuristic #5: Error prevention

#	Review Checklist	Yes	No	N/A	Comments
5.1	Even better than a good error message is a careful design that prevents the	☐	☐	☐	

	problem from occurring in the first place. Does the app eliminate error-prone conditions or check for them and give users a confirmation option before they take action?		
5.2	Are menu choices logical, distinctive, and mutually exclusive?	☐ ☐ ☐	
5.3	Are data inputs case-blind whenever possible?	☐ ☐ ☐	
5.4	Does the system warn users if they are about to make a potentially serious error?	☐ ☐ ☐	
5.5	Do the data entry screens and dialog boxes show the number of character spaces available in a field?	☐ ☐ ☐	
5.6	Do fields in data entry screens and dialog boxes contain default values if necessary?	☐ ☐ ☐	
5.7	Is accidental activation avoided or foreseen (a back button is offered)?	☐ ☐ ☐	
5.8	Is the touchable area large enough (Research has shown that the best target size for widgets is 1cmx1cm for touch devices)?	☐ ☐ ☐	
5.9	Is target crowding avoided (When targets are placed too close to each other, users can easily hit the wrong target)?	☐ ☐ ☐	
5.10	Even though the visible portion of the target may be small, is there an invisible target space so that if the user hits that space, the hits will still count?	☐ ☐ ☐	
5.11	When multiple items are listed in a column, one above the other, can the user press anywhere in the row to select the target that corresponds to that row?	☐ ☐ ☐	
5.12	Is downloading software that is inappropriate for the user phone avoided?	☐ ☐ ☐	
5.13	Are JavaScript and Flash use avoided?	☐ ☐ ☐	
5.14	Is confirmation from the user taken before performing, executing, or using complex procedures to avoid unintentional errors?	☐ ☐ ☐	

5.15	Are unavailable features hidden or disabled?	☐	☐	☐	
5.16	Are users warned about critical actions and granted access to additional information?	☐	☐	☐	

Heuristic #6: Help users recognize, diagnose, and recover from errors

#	Review Checklist	Yes	No	N/A	Comments
6.1	Are error messages expressed in plain language (no codes) familiar language of the user?	☐	☐	☐	
6.2	Do the error messages correctly pinpoint the problem and constructively suggest a solution?	☐	☐	☐	
6.3	When signaling an input error in a form, is the text box that needs to be changed specially marked or is the text highlighted in it?	☐	☐	☐	
6.4	Due to limited data entry and, consequently, higher user error rate, does the phone provide meaningful feedback? Short error message? Easy error restore and recovery options?	☐	☐	☐	

Heuristic #7: Recognition rather than recall

#	Review Checklist	Yes	No	N/A	Comments
7.1	Are instructions for using the system visible or easily retrieved when needed?	☐	☐	☐	
7.2	Is high levels of concentration unnecessary, and remembering information no longer than two to fifteen seconds?	☐	☐	☐	
7.3	Are all data a user needs on display at each step in a transaction sequence?	☐	☐	☐	
7.4	If the user has to navigate between multiple screens, does the system use context labels, menu maps, and placemarks as navigational aids?	☐	☐	☐	
7.5	After the user completes an action (or group of actions), does the feedback indicate that the next group of actions can be started?	☐	☐	☐	

7.6	Are optional data entry fields clearly marked?	☐	☐	☐	
7.7	Do data entry screens and dialogue boxes show when fields are optional?	☐	☐	☐	
7.8	Is page length controlled?	☐	☐	☐	
7.9	Should the task flow begin with the actions that are important to the main task? And can the user start the task as soon as possible?	☐	☐	☐	
7.10	Are the controls associated with the task grouped together and reflect the sequence of actions in the task?	☐	☐	☐	
7.11	For a question and answer interface, are visual cues and spaces used to differentiate questions, hints, instructions, and user input?	☐	☐	☐	
7.12	Does the data display start in the upper-left corner of the screen?	☐	☐	☐	
7.13	Are commands formatted using spaces, justifications, and visual cues to make scanning easier?	☐	☐	☐	
7.14	Do text areas have "breathing space" around them?	☐	☐	☐	
7.15	Are there "white" areas between information objects for visual relaxation?	☐	☐	☐	
7.16	Does the system provide visibility: i.e., by viewing, can the user tell the state of the system and alternative actions?	☐	☐	☐	
7.17	Are size, bold, underline, color, shading, or typography used to indicate the quantity or relative importance of different screen items?	☐	☐	☐	
7.18	Is color used in conjunction with some other redundant cue?	☐	☐	☐	
7.19	Is there good color contrast and brightness between the image and the background color?	☐	☐	☐	
7.20	Have light, bright, and saturated colors been used to emphasize the data and have darker, duller, and desaturated colors been used to reduce the emphasis of the data?	☐	☐	☐	

7.21	Is the visual page space well used?	☐	☐	☐	
7.22	On data entry screens and dialog boxes, are dependent fields shown only when needed?	☐	☐	☐	
7.23	Are field labels close to fields, but separated by at least one space?	☐	☐	☐	
7.24	Is the first word of each menu choice the most important?	☐	☐	☐	
7.25	Are inactive menu items greyed out or omitted?	☐	☐	☐	
7.26	Are there menu selection defaults?	☐	☐	☐	
7.27	Is there a clear visual difference between the "choose one" and "choose many" menus?	☐	☐	☐	
7.28	Is there a breadcrumb on a site with a deep navigation structure (many navigation branches)? And, is it avoided on sites with shallow navigation structures?	☐	☐	☐	
7.29	Does the device provide visible objects, actions, and options to prevent users from remembering information from one part of the dialog box to another?	☐	☐	☐	
7.30	Are objects, actions, and options visible to the user to minimize their memory load?	☐	☐	☐	

Heuristic #8: Flexibility and efficiency of use

#	Review Checklist	Yes	No	N/A	Comments
8.1	Accelerators are usually invisible to novice users but can often speed up interaction for expert users. Are accelerators used?	☐	☐	☐	
8.2	Is the searching box easily accessible?	☐	☐	☐	
8.3	Is the searching box easily recognizable?	☐	☐	☐	
8.4	Is there any advanced search option?	☐	☐	☐	
8.5	Are search results displayed comprehensively to users?	☐	☐	☐	
8.6	Is the box width appropriated?	☐	☐	☐	
8.7	Is the user assisted if the search results are impossible to calculate?	☐	☐	☐	

8.8	Is there a search box on the mobile site homepage?	☐	☐	☐	
8.9	Is the search box length at least the size of the average search string? Or better, is it the largest size that will fit on the screen?	☐	☐	☐	
8.10	Is the search string preserved between searches? Are there autocompletes and suggestions?	☐	☐	☐	
8.11	Are multiple search boxes with different functions on the same page avoided?	☐	☐	☐	
8.12	If the search returns no results, is some alternative search offered or a link to the search results on the full page?	☐	☐	☐	
8.13	Are links with a good smell of information (that is, links that clearly indicate where they are taking the user)?	☐	☐	☐	
8.14	Are there links to related content to help users navigate faster between similar topics?	☐	☐	☐	
8.15	Does the device charge fast?	☐	☐	☐	
8.16	Is the device ease to learn?	☐	☐	☐	
8.17	To improve interaction efficiency, does the device provide an alternative way to display the same functionality as a shortcut?	☐	☐	☐	
8.18	Are all features found in a single interaction?	☐	☐	☐	
8.19	If possible, does the system suggest support and provide customization options for frequently performed actions?	☐	☐	☐	
8.20	Does the device provide basic and advanced settings facilities to set and customize shortcuts for frequently performed actions?	☐	☐	☐	
8.21	Is the information loaded and displayed in a reasonable time?	☐	☐	☐	
8.22	Are a number of steps required to perform a task minimally?	☐	☐	☐	
8.23	Are animations and transitions displayed seamlessly?	☐	☐	☐	

Heuristic #9: Aesthetic and minimalist design

#	Review Checklist	Yes	No	N/A	Comments
9.1	Each additional unit of information in the dialog competes with the relevant unit of information and reduces its relative visibility. Does the dialog contain relevant and frequently needed information?	☐	☐	☐	
9.2	Is the time to reach the target a function of the distance and the size of the target according to Fitts' Law?	☐	☐	☐	
9.3	Is only (and all) information important for decision making shown on the screen?	☐	☐	☐	
9.4	Are field labels brief, familiar, and descriptive?	☐	☐	☐	
9.5	Are the prompts expressed in the affirmative, and do they use active voice?	☐	☐	☐	
9.6	Is the layout clearly designed to avoid visual noise?	☐	☐	☐	
9.7	Are the app icons recognizable enough to be found in a crowded list of apps?	☐	☐	☐	
9.8	Does the use of images and multimedia content add value?	☐	☐	☐	
9.9	Is the image a good size? Are they understandable? Is the resolution appropriate?	☐	☐	☐	
9.10	Are cyclical animations avoided?	☐	☐	☐	
9.11	Is flash content avoided?	☐	☐	☐	
9.12	Should the use of animated carousels be avoided? And if there is, can the user control it?	☐	☐	☐	
9.13	Is the image size smaller than the screen (The whole image must be viewable without scrolling)?	☐	☐	☐	
9.14	For cases where a customer may need access to a higher resolution image, is the screen size image initially displayed, and is there a separate link to the higher resolution variant?	☐	☐	☐	

9.15	When using thumbnails, can users tell what they are about?	☐	☐	☐	
9.16	Do captions help understand the meaning of images that are part of the article if the meaning is not clear from the context of the article?	☐	☐	☐	
9.17	Are moving animation avoided?	☐	☐	☐	
9.18	When using a video, is there a textual description of the video?	☐	☐	☐	
9.19	Do clicking thumbnails and clicking video titles play the same video?	☐	☐	☐	
9.20	Is the video length indicated?	☐	☐	☐	
9.21	If the video does not play on the user's device, what is the message that is displayed with this information?	☐	☐	☐	
9.22	Is the entire surface of the screen used to efficiently place information (especially for popovers and modals)?	☐	☐	☐	
9.23	Has excessive detail in icon design been avoided?	☐	☐	☐	
9.24	Are each individual icon a member of a harmonious icon family?	☐	☐	☐	
9.25	Does each icon stand out from its background?	☐	☐	☐	
9.26	Are all icons in a set visually and conceptually distinct?	☐	☐	☐	
9.27	Is each lower level menu option related to only one higher level menu?	☐	☐	☐	
9.28	Are menu titles brief, yet long enough to communicate?	☐	☐	☐	
9.29	Desktop websites have strong guidelines for avoiding horizontal scrolling. But for touchscreens, horizontal swipes are often fine. Does this option count?	☐	☐	☐	
9.30	Is the site designed to avoid a large number of persistent navigation options across all pages?	☐	☐	☐	
9.31	Is the information presented in a consistent manner and divided into small sections? And easy to find to minimize the user's cognitive load?	☐	☐	☐	
9.32	To access the full version of the app, does the phone allow users to access the	☐	☐	☐	

	desktop version of the site from their phone?		
9.33	Is the screen area partially used by a keyboard?	☐ ☐ ☐	
9.34	The most relevant information should be highlighted visually through larger sizes, colors, use of markers, etc. Is the information highlighted that way visually?	☐ ☐ ☐	
9.35	Due to the screen size limit, unlike websites for desktop, the navigation is NOT repeated on all pages?	☐ ☐ ☐	
9.36	Is the display of unwanted information avoided to overload the screen?	☐ ☐ ☐	

Heuristic #10: Help and documentation

#	Review Checklist	Yes	No	N/A	Comments
10.1	Although assistance and documentation are required as part of the system, is the system in a usable condition without documentation support?	☐	☐	☐	
10.2	Is the information in the help and documentation easy to find, focused on the user's task, listed concrete steps to be taken, and not too large?	☐	☐	☐	
10.3	Are on-line instructions visually distinct?	☐	☐	☐	
10.4	Do the instructions follow the sequence of user actions?	☐	☐	☐	
10.5	If menu choices are ambiguous, does the system provide additional explanatory information when an item is selected?	☐	☐	☐	
10.6	If a menu item is ambiguous, does the system provide additional explanatory information when the item is selected?	☐	☐	☐	
10.7	Is the help function visible, for example, a button labeled HELP or a custom menu?	☐	☐	☐	
10.8	Is the help system interface (navigation, presentation, and conversation) consistent with the navigation,	☐	☐	☐	

	presentation, and conversation interfaces of the applications it supports?		
10.9	Is the information easy to find?	☐ ☐ ☐	
10.10	Is the visual layout well designed?	☐ ☐ ☐	
10.11	Is the information accurate, complete, and understandable?	☐ ☐ ☐	
10.12	Is the information relevant? It should be relevant in the following aspects: Goal-oriented (What can I do with this program?), Descriptive (What is this for?), Procedural (How do I do this task?), Interpretive (Why did it happen??) and Navigation (Where am I?).<br>10.13 Is there context sensitive help?	☐ ☐ ☐	
10.13	Is there context-sensitive help?	☐ ☐ ☐	
10.14	Can the user change the level of detail available?	☐ ☐ ☐	
10.15	Can users easily switch between help and their work?	☐ ☐ ☐	
10.16	Is it easy to access and return from the help system?	☐ ☐ ☐	
10.17	Can users continue work where they left off after accessing help?	☐ ☐ ☐	
10.18	If there is an FAQ section, is the selection and editorial of questions and answers correct?	☐ ☐ ☐	
10.19	Does the design focus on one feature at a time? (Only the instructions required for the user to start should be presented at one time).	☐ ☐ ☐	
10.20	Does the app provide help options where common problems and ways to solve them are specified?	☐ ☐ ☐	
10.21	Are issues considered in the help option easy to find?	☐ ☐ ☐	
10.22	Does the device provide easy-to-find and helpful documentation, focus on the user's current task and show concrete steps to follow?	☐ ☐ ☐	

Heuristic #11: Privacy

#	Review Checklist	Yes	No	N/A	Comments
11.1	Are protected areas completely inaccessible?	☐	☐	☐	
11.2	Are protected or secret areas only accessible with a certain password?	☐	☐	☐	
11.3	Is there any information about how personal data is protected and about content copyright?	☐	☐	☐	
11.4	For multiuser devices: Is permanent login in the app avoided?	☐	☐	☐	
11.5	If the app stores credit card info, can users decide if they want to stay signed in? If the user chooses to stay logged in, he/she should get a message informing him of the possible risk.	☐	☐	☐	
11.6	Is the user's confidential data protected by the device?	☐	☐	☐	

Heuristic #12: Skills

#	Review Checklist	Yes	No	N/A	Comments
12.1	Is the word "default" avoided and replaced with "Standard", "Use Custom Settings", "Restore Initial Settings", or some other more specific term that describes what is actually going to happen?	☐	☐	☐	
12.2	If the system supports both novice and expert users, is some level of error message detail available?	☐	☐	☐	
12.3	If the system supports both novice and expert users, are different levels of detail available?	☐	☐	☐	
12.4	Are users the initiators of actions rather than the responders?	☐	☐	☐	
12.5	Do the selected input device(s) match user capabilities?	☐	☐	☐	
12.6	Are important keys (for example, ENTER, TAB) larger than other keys?	☐	☐	☐	
12.7	Does the system correctly anticipate and prompt the next possible user activity?	☐	☐	☐	

Heuristic #13: Pleasurable interaction

#	Review Checklist	Yes	No	N/A	Comments
13.1	Is the user's job protected? For example, for data entry screens with many fields or where the original may be incomplete, can the user save the partially filled screen?	☐	☐	☐	
13.2	Do the selected input device(s) match environmental constraints?	☐	☐	☐	
13.3	Are typing requirements minimal for question and answer interfaces?	☐	☐	☐	
13.4	Does the system resolve unambiguous partial input on data entry fields?	☐	☐	☐	
13.5	Users don't like typing. Does the information count for the user? For example, ask for only postal codes and calculate states and cities; may offer a list of cities if there are more under the same zip code	☐	☐	☐	
13.6	Is the input data tolerant of typos and offers correction? (Don't make the user type in incomplete information. For example, accept "123 Main" instead of "123 Main St.")	☐	☐	☐	
13.7	Can users save history and select previously typed info?	☐	☐	☐	
13.8	Does default information make sense to the user?	☐	☐	☐	
13.9	If the app doesn't store any sensitive information (e.g., credit cards), does the user stay logged in (with logout prominently displayed)?	☐	☐	☐	
13.10	Is the number of submissions (and clicks) minimized for users to enter information on the site?	☐	☐	☐	
13.11	When logging in is required, is the graphical password used at least a few times, to avoid typing?	☐	☐	☐	
13.12	Is registration not mandatory? Is skipping registration the default option?	☐	☐	☐	
13.13	When login is required, is there an option that allows the user to see the password clearly?	☐	☐	☐	

13.14	When a product list is presented, are the image thumbnails large enough for the user to get some information out of it?	☐ ☐ ☐	
13.15	On the product page, does the image size fit the screen? Are there links to higher resolution images where the product requires closer inspection?	☐ ☐ ☐	
13.16	Is there the option to email a product to a friend?	☐ ☐ ☐	
13.17	Is there the option to save the product in a wish list?	☐ ☐ ☐	
13.18	On an ecommerce site, are important links included on the homepage to the following information: — location and opening hours (if applicable), — shipping costs, — phone number, — order status, and — promotions or products by event?	☐ ☐ ☐	
13.19	Whenever a user makes a transaction on the phone, can they save the confirmation number for the transaction by emailing themselves? If the phone has an embedded screenshot feature, are there any instructions on how to take a screenshot of it?	☐ ☐ ☐	
13.20	Does the device provide a pleasant iteration with the user so that the user does not feel uncomfortable while using the app?	☐ ☐ ☐	

References :

1. Nielsen J. 10 Usability Heuristics for User Interface Design. 1994 Apr 24. URL: http://www.nngroup.com/articles/ ten-usability-heuristics/ [accessed 2021-11-25]
2. Khowaja, K., & Al-Thani, D., 2020. New checklist for the heuristic evaluation of mHealth apps (HE4EH): Development and usability study. *JMIR mHealth uHealth* 8. doi:10.2196/20353
