# Supplementary material for: Integrated Diabetes Self-Management (IDSM) mobile application to improve self-management and glycemic control among patients with Type 2 Diabetes Mellitus (T2DM) in Indonesia: A mixed methods study protocol
Source: PLoS One. 2022 Nov 28;17(11):e0277127. doi: 10.1371/journal.pone.0277127 (PMC9704669; doi:10.1371/journal.pone.0277127)
Supplement: S7 File — (PDF) [file pone.0277127.s007.pdf]

THINK ALOUD GUIDELINE

A. Setting

1. Make sure that the setting is such that the subject feels at ease.
2. The subject should be settled comfortably.
3. The room should be quiet, a glass of water should be at hand, the chair should be comfortable.
4. The situation should be focused on the task and minimize distractions to thought processes as much as possible to avoid their influencing its course

B. Opening

1. The researcher introduces himself
2. Participants will be given an explanation and informed consent
3. Participants will be given an explanation about the purpose of the study, about what will happen and about data protection.
4. Explaining that data will be handled strictly confidential.
5. Write down the instructions beforehand and read them to the subject

C. Instruction

1. Instructions about the task at hand should be given as customary
2. Participants will be given a task in accordance to their role.

D. Warming Up

1. Give the subject an opportunity to practice thinking aloud to warming up
2. Give them a few minutes to a quarter of an hour to do a little training.

E. Implementation of Think Aloud

1. Participants are asked to perform the task and say out loud what they are doing, seeing, and thinking while they are using the apps to complete the given task.

2. The researcher should prompt the subject by just, and only just saying: 'Keep on talking'.

3. Correcting or assisting participants in carrying out their task should be avoided.

F. Recording

1. All of the behaviour and words said by the participants will be audio visually recorded. Including the screen of the app.
2. Includes instructions and practice phases, to be recorded for checking after that whether the procedure is done correctly.
3. Always check the recording device and check regularly during the session.

G. Transcription of the protocol

1. After the session has been recorded, it has to be transcribed
2. Write down the observed data separately on a special note
3. Unreasonable interpretations should be avoided

H. Review

1. Review the protocol with the subject as soon as possible after the actual thinking session

Reference :

1. Someren, M.W. van, Barnard, Y.F., & Sandberg, J.A.C., 1995. The think aloud method; A practical guide to modelling cognitive processes, Information Processing & Management.
