# Supplementary material for: Integrated Diabetes Self-Management (IDSM) mobile application to improve self-management and glycemic control among patients with Type 2 Diabetes Mellitus (T2DM) in Indonesia: A mixed methods study protocol
Source: PLoS One. 2022 Nov 28;17(11):e0277127. doi: 10.1371/journal.pone.0277127 (PMC9704669; doi:10.1371/journal.pone.0277127)
Supplement: S8 File — (PDF) [file pone.0277127.s008.pdf]

Indonesian Version- Diabetes Self-Management Instrument (IDN-DSMI 35)

Petunjuk Pengisian:

Pernyataan-pernyataan dibawah ini tentang bagaimana yang anda rasakan selama 3 bulan terakhir. Tidak ada jawaban benar atau salah. Silahkan memberikan tanda "X" pada kolom yang sesuai selama menderita diabetes. Nomor disamping berarti :

1 : Tidak pernah (tidak akan terjadi)
2 : Kadang-kadang (terjadi 1-3 hari setiap minggu; atau terjadi kurang dari setengah dalam seminggu)
3 : Biasanya (terjadi 4-6 hari setiap minggu; atau terjadi lebih dari setengah dalam seminggu)
4 : Selalu (terjadi setiap hari)

No	**Pernyataan Yang Dirasakan**	**Selalu**	**Biasanya**	**Kadang -kadang**	**Tidak Pernah**
1.	Ketika memilih makanan dan porsi yang saya makan, saya mempertimbangkan efek pada kadar gula darah saya	☐4	☐3	☐2	☐1
2.	Saya dapat berpartisipasi dalam aktivitas sosial dan tetap mengatur/mengontrol diabetes saya	☐3	☐2	☐1	☐0
3.	Saya tahu bagaimana mengatur porsi dan memilih makanan ketika makan di luar	☐3	☐2	☐1	☐0
4.	Saya menganggap bahwa management diabetes sebagai cara untuk tetap hidup dengan sehat	☐3	☐2	☐1	☐0
5.	Saya merasa nyaman bertanya pada penderita diabetes lain tentang tips dalam mengontrol diabetes	☐3	☐2	☐1	☐0
6.	Gaya hidup sehari-hari saya sekarang lebih sehat daripada sebelum saya menderita diabetes	☐3	☐2	☐1	☐0
7.	Saya telah sukses menyatukan diabetes kedalam kehidupan sehari-hari saya	☐3	☐2	☐1	☐0
8.	Saya memperhatikan sinyal yang diberikan oleh tubuh saya berhubungan dengan kadar gula darah	☐3	☐2	☐1	☐0
9.	Saya memperhatikan terhadap situasi kehidupan sehari-hari saya yang mungkin menyebabkan kadar gula darah saya berubah	☐3	☐2	☐1	☐0
10.	Saya dapat mengenali tanda dan gejala yang umum berhubungan dengan kadar gula darah	☐3	☐2	☐1	☐0
11.	Saya dapat memahami alasan pada perubahan pada kadar gula darah	☐3	☐2	☐1	☐0
12.	Saya membandingkan perbedaaan antara kadar gula darah saat ini dengan kadar gula darah target saya	☐3	☐2	☐1	☐0

13.	Saya memonitor kemajuan saya dengan melihat kadar gula darah dan kadar A1c	☐3	☐2	☐1	☐0
14.	Saya mengambil tindakan berdasarkan sinyal tubuh seperti haus, kehilangan kesabaran, dan perasaan cemas	☐3	☐2	☐1	☐0
15.	Ketika saya merasa kadar gula darah saya terlalu rendah, saya mengecek kadar gula darah sesegera mungkin	☐3	☐2	☐1	☐0
16.	Saya menentukan tindakan apa yang saya ambil berdasarkan hasil dari tindakan saya sebelumnya	☐3	☐2	☐1	☐0
17.	Ketika saya merasa tidak sehat tetapi saya tidak yakin apakah ini disebabkan karena kadar gula darah yang tinggi atau rendah, saya mengecek kadar gula darah sesegera mungkin	☐3	☐2	☐1	☐0
18.	Saya dapat menyesuaikan rutinitas diabetes saya supaya sesuai dengan situasi yang baru (misalnya jauh dari rumah, merubah jadwal saya, dan menghadiri perayaan/pesta)	☐3	☐2	☐1	☐0
19.	Ketika saya merasa kadar gula darah saya terlalu tinggi, saya mengecek kadar gula darah sesegera mungkin	☐3	☐2	☐1	☐0
20.	Saya nyaman bertanya kepada petugas kesehatan (dokter, perawat, dll) tentang bagaimana rencana perawatan saya	☐3	☐2	☐1	☐0
21.	Saya bekerja bersama dengan petugas kesehatan (dokter, perawat, dll) saya untuk mengidentifikasi kemungkinan penyebab kontrol diabetes saya jelek	☐3	☐2	☐1	☐0
22.	Saya nyaman menceritakan kepada petugas kesehatan (dokter, perawat, dll) saya seberapa fleksibel saya ingin mengikuti rencana perawatan saya.	☐3	☐2	☐1	☐0
23.	Saya nyaman menceritakan kepada petugas kesehatan (dokter, perawat, dll) saya tentang perubahan yang ingin saya buat dalam rencana perawatan saya	☐3	☐2	☐1	☐0
24.	Saya bercerita kepada orang lain (misal: teman-teman, keluarga saya) tentang situasi dimana saya memerlukan bantuan mereka untuk mengontrol diabetes saya	☐3	☐2	☐1	☐0
25.	Saya nyaman berdiskusi tentang hasil test gula darah saya dengan petugas kesehatan (dokter, perawat, dll) saya	☐3	☐2	☐1	☐0

26.	Saya meminta (misal: teman-teman, keluarga saya) untuk membantu saya dalam reaksi terhadap gula darah yang tinggi jika diperlukan	☐3	☐2	☐1	☐0
27.	Saya nyaman bertanya kepada petugas kesehatan (dokter, perawat, dll) saya tentang sumber-sumber yang dapat membantu saya mengatur diabetes saya	☐3	☐2	☐1	☐0
28.	Saya mengecek kadar gula darah untuk membantu membuat keputusan perawatan sendiri (misal: pengobatan, diet, dan olah raga)	☐3	☐2	☐1	☐0
29.	Saya mengatur pemilihan makanan untuk membantu mengontrol gula sarah dan berat badan saya	☐3	☐2	☐1	☐0
30.	Saya minum obat diabetes sesuai dengan resep	☐3	☐2	☐1	☐0
31.	Saya berolah raga cukup untuk membantu mengontrol kadar gula darah dan berat badan saya	☐3	☐2	☐1	☐0
32.	Saya menjaga berat badan dalam rentang yang ditetapkan oleh petugas kesehatan (dokter, perawat, dll) dan saya	☐3	☐2	☐1	☐0
33.	Saya menemui petugas kesehatan (dokter, perawat, dll) saya setiap 1-3 bulan	☐3	☐2	☐1	☐0
34.	Jika saya mengalami reaksi kadar gula darah yang rendah saya tahu bagaimana menanganinya	☐3	☐2	☐1	☐0
35.	Saya minum sejumlah obat diabetes yang telah diresepkan untuk saya	☐3	☐2	☐1	☐0

Reference :

1. Rahayu, H.T., & Chen, C.M., 2020. Psychometric Testing of an Indonesian-Version Diabetes Self-Management Instrument. *J. Nurs. Res.* 28. doi:10.1097/jnr.0000000000000403
