# Supplementary material for: Integrated Diabetes Self-Management (IDSM) mobile application to improve self-management and glycemic control among patients with Type 2 Diabetes Mellitus (T2DM) in Indonesia: A mixed methods study protocol
Source: PLoS One. 2022 Nov 28;17(11):e0277127. doi: 10.1371/journal.pone.0277127 (PMC9704669; doi:10.1371/journal.pone.0277127)
Supplement: Attachment — Submitted filename: Respons To Reviewers_R2.pdf [file pone.0277127.s010.pdf]

RESPONS TO REVIEWERS

<table>
<tr><th>No</th><th>Comments</th><th>Respons</th></tr>
<tr><td></td><td>Journal Requirements:

Please review your reference list to ensure that it is complete and correct. If you have cited papers that have been retracted, please include the rationale for doing so in the manuscript text, or remove these references and replace them with relevant current references. Any changes to the reference list should be mentioned in the rebuttal letter that accompanies your revised manuscript. If you need to cite a retracted article, indicate the article's retracted status in the References list and also include a citation and full reference for the retraction notice.</td><td>Thank you for the suggestion.

Removed References :
Hou C, Xu Q, Diao S, Hewitt J, Li J, Carter B. Mobile phone applications and self-management of diabetes: A systematic review with meta-analysis, meta-regression of 21 randomized trials and GRADE. Diabetes, Obes Metab. 2018;20(8):2009–13.
(This reference is not used to determine the standard deviation and mean in the calculation of the sample because the result is not correct).

Additional References :
Pahlevi R. Jumlah Penderita Diabetes Indonesia Terbesar Kelima di Dunia. Artikel. 2021;1. (1)
(This reference is used to add data on the number of patient with diabetes melitus in Indonesia in 2021 on page 3 line 54 and page 19 line 439 – 440).

Lorig K R, Holman H R. Self Management Education: History, Definition, Outcomes and Mechanisms. Ann Behav Med [Internet]. 2003;26(1):1–7. Available from: http://www.springerlink.com/index/96t1713736v23t27.pdf (7)
(This reference explains the definition of self-management on page 3 line 71 and page 19 line 454 – 456)

Hasanah N, Ikawati Z, Zainal ZA. The effectiveness of smartphone application-based education “teman diabetes” on clinical outcomes of type-2 diabetes mellitus patients. Res J Pharm Technol. 2021;14(7):3625–30. (26)
(This reference explains the standard deviation and mean difference in previous studies which were used to determine the sample size on page 9 line 222 and page 21 line 507 – 509).

Lally P, Gardner B. Promoting habit formation. Health Psychol Rev. 2013;7(SUPPL1):37–41. (29)
(This reference describes the change in behavior into a habit that occurs for 21 days. This is the basis for conducting self-management evaluations monthly on page 10 line 241 and page 22 line 514 – 515).

Al Amin M, Juniati D. Klasifikasi Kelompok Umur Manusia Berdasarkan Analisis Dimensi. J Ilm Mat. 2017;2(6):1–10. (30)</td></tr>
</table>

		(This reference is used as a literature for determining the age 21 of adult participants who are included as inclusion criteria on page 11 line 255 and page 22 line 516 – 517).<br><br>Cohen J. Statistical Power Analysis for the Behavioral Sciences. Statistical Power Analysis for the Behavioral Sciences. 2013. (33)<br>(This reference is used as a reference for Cohen's formula in the effect size analysis on page 14 line 332 and page 22 line 522 – 523).
	**Additional Editor Comments:**<br><br>The authors improved the manuscript. However, it is necessary to consider the following concerns;<br>• Authors should calculate the sample size considering the "two sided" test. The population is relatively small.<br>• The authors mentioned that the control group will receive standard treatment via WhatsApp chat. Is this a standard treatment in Indonesia? How frequently the nurses will send messages to the participants? Is that a group based chat or personal communication will be allowed? The treatment for control group should be given in detail.<br>• The age range of inclusion criteria should be reconsidered. How do you discriminate a Type 2 and Type 1 or Moody Type DM in a participant aged 18 years?	Thank you for the suggestion.<br><br>• Sample size have calculate considering the "two sided" test, and it results 35 participants. To anticipate the possibility of participants dropping out, a number of samples will be added to keep them fulfilled by the formula : n' = n/(1-f) so the sample to be used is 39 participants for each group (intervention and control group). (page 9 line 221).<br><br>• Regarding the use of whatsapp chat, patients may have personal communication with health provider at PHC in addition to group communication. They communicate via WA chat as needed. Whatsapp is very popular among Indonesian, most of Indonesian people who has smartphone using whatsapp including patients with diabetes. So it is easy for a health provider to communicate with all patients who are in Prolanis program using whatsapp, eventhough it is not written national standar.<br>The control group will use the whatsapp group to communicate or consult directly with a health provider. Self-management will be carried out independently by the patient in his or her home. Monitoring by a health provider will be carried out using whatsapp group and when patient come to the PHC for routine check up. (page 9 line 214 – 218).<br><br>• The age range for the inclusion criteria is participants aged 21 years old and older who have no history of diabetes since childhood. (page 11 line 254 , 256-257).
	**Comments to the Author**	

1	Does the manuscript provide a valid rationale for the proposed study, with clearly identified and justified research questions?<br><br>The research question outlined is expected to address a valid academic problem or topic and contribute to the base of knowledge in the field.<br><br>Reviewer #1: Partly<br><br>Reviewer #2: Yes	Thank you for the suggestion.<br>Research questions have answered valid academic topics or problems based on knowledge in the field.
2	Is the protocol technically sound and planned in a manner that will lead to a meaningful outcome and allow testing the stated hypotheses?<br><br>The manuscript should describe the methods in sufficient detail to prevent undisclosed flexibility in the experimental procedure or analysis pipeline, including sufficient outcome-neutral conditions (e.g. necessary controls, absence of floor or ceiling effects) to test the proposed hypotheses and a statistical power analysis where applicable. As there may be aspects of the methodology and analysis which can only be refined once the work is undertaken, authors should outline potential assumptions and explicitly describe what aspects of the proposed analyses, if any, are exploratory.<br><br>Reviewer #1: Partly<br><br>Reviewer #2: Yes	Thank you for the consideration.<br>We explain the analysis statistic and the alternative analysis when there are data is normally distributed or not.<br>Before the data is analyzed, it is necessary to test the normality of the data, to find out whether the data is normally distributed or not. If the data are normally distributed, then we use the parametric statistical analysis as planned. However, if the data is not normally distributed, we have also included an alternative statistical analysis to be carried out. Regarding alternative analysis statistic we already mention in detail on page 14 line 318 – 328.
3	Is the methodology feasible and described in sufficient detail to allow the work to be replicable?<br><br>Descriptions of methods and materials in the protocol should be reported in sufficient detail for	Thank you for the consideration.<br><br>Descriptions of methods and materials in the protocol will be reported in sufficient detail for another researcher to reproduce all experiments and analyses. The protocol have described the appropriate controls, sample size

	another researcher to reproduce all experiments and analyses. The protocol should describe the appropriate controls, sample size calculations, and replication needed to ensure that the data are robust and reproducible.<br><br>Reviewer #1: Yes<br><br>Reviewer #2: Yes	calculations, and replication needed to ensure that the data are robust and reproducible.
4	Have the authors described where all data underlying the findings will be made available when the study is complete?<br><br>The PLOS Data policy requires authors to make all data underlying the findings described in their manuscript fully available without restriction, with rare exception, at the time of publication. The data should be provided as part of the manuscript or its supporting information, or deposited to a public repository. For example, in addition to summary statistics, the data points behind means, medians and variance measures should be available. If there are restrictions on publicly sharing data—e.g. participant privacy or use of data from a third party—those must be specified.<br><br>Reviewer #1: No<br><br>Reviewer #2: Yes	Thank you for the suggestion.<br><br>This study will provide opportunities for other researchers who need the same data by contacting the corresponding author. Dissemination of findings and data sharing will be done after this research is completed. The data will be provided as part of the manuscript. Once we finished with data collection.<br>To facilitate data access and data protection during the research process, we will report any research progress to all teams regularly. This study's results will be published in reputable international journals. (Page 15 line 346 – 349 and page 17 line 398 - 400)
5	Is the manuscript presented in an intelligible fashion and written in standard English?<br><br>PLOS ONE does not copyedit accepted manuscripts, so the language in submitted articles must be clear, correct, and unambiguous. Any typographical or grammatical errors should be corrected at revision, so please note any specific errors here.	Thank you for the consideration.<br><br>The manuscript has been presented in understandable standard English.

	Reviewer #1: Yes<br><br>Reviewer #2: Yes	
6	Review Comments to the Author<br><br>Please use the space provided to explain your answers to the questions above and, if applicable, provide comments about issues authors must address before this protocol can be accepted for publication. You may also include additional comments for the author, including concerns about research or publication ethics.<br><br>You may also provide optional suggestions and comments to authors that they might find helpful in planning their study.<br><br>(Please upload your review as an attachment if it exceeds 20,000 characters)<br><br>Reviewer #1:<br>The authors improved the manuscript. However, the following revisions are needed for better explaining;<br>• Authors should calculate the sample size considering the "two sided" test. The population is relatively small.<br><br>• The authors mentioned that the control group will receive standard treatment via Whatsapp chat. Is this a standard treatment in Indenosia? How frequently the nurses will send messages to the participants? Is that a group based chat or personal comminucation will be allowed?	Thank you for the suggestion.<br><br>• Sample size have calculate considering the "two sided" test, and it results 35 participants. To anticipate the possibility of participants dropping out, a number of samples will be added to keep them fulfilled by the formula : n' = n/(1-f) so the sample to be used is 39 participants for each group (intervention and control group). (page 9 line 221).<br><br>• Regarding the use of whatsapp chat, patients may have personal communication with health provider at PHC in addition to group communication. They communicate via WA chat as needed. Whatsapp is very popular among Indonesian, most of Indonesian people who has smartphone using whatsapp including patients with diabetes. So it is easy for a health provider to communicate with all patients who are in Prolanis

	The treatment for control group should be given in detail.<br><br>• The age range of inclusion criteria should be reconsidered. How do you discriminate a Type 2 and Type 1 or Moody Type DM in a participant aged 18 years?<br><br>Reviewer #2:<br>Thank you for addressing my comments. This study now sound very clear and can be published as it is.	program using whatsapp, eventhough it is not written national standar.<br>The control group will use the whatsapp group to communicate or consult directly with a health provider. Self-management will be carried out independently by the patient in his or her home. Monitoring by a health provider will be carried out using whatsapp group and when patient come to the PHC for routine check up. (page 9 line 214 – 218).<br><br>• The age range for the inclusion criteria is participants aged 21 years old and older who have no history of diabetes since childhood. (page 11 line 254 , 256-257).<br><br>Thank you for the support.
	While revising your submission, please upload your figure files to the Preflight Analysis and Conversion Engine (PACE) digital diagnostic tool, https://pacev2.apexcovantage.com/. PACE helps ensure that figures meet PLOS requirements. To use PACE, you must first register as a user. Registration is free. Then, login and navigate to the UPLOAD tab, where you will find detailed instructions on how to use the tool. If you encounter any issues or have any questions when using PACE, please email PLOS at figures@plos.org. Please note that Supporting Information files do not need this step.	Thank you for the suggestion.<br>Figures 1 and 2 (S9 and S10) have been created according to the PACE format and are in accordance with the PACE procedure.
