# Supplementary material for: Integrated Diabetes Self-Management (IDSM) mobile application to improve self-management and glycemic control among patients with Type 2 Diabetes Mellitus (T2DM) in Indonesia: A mixed methods study protocol
Source: PLoS One. 2022 Nov 28;17(11):e0277127. doi: 10.1371/journal.pone.0277127 (PMC9704669; doi:10.1371/journal.pone.0277127)
Supplement: Attachment — Submitted filename: Response to Reviewers.docx [file pone.0277127.s009.docx]

**HASIL REVISI ARTIKEL JURNAL PLOS ONE**

**Comments to the Author**

| No | Comment | Respon |
| --- | --- | --- |
| 1 | Does the manuscript provide a valid rationale for the proposed study, with clearly identified and justified research questions?  The research question outlined is expected to address a valid academic problem or topic and contribute to the base of knowledge in the field.  Reviewer #1: Partly  Reviewer #2: Yes | Thank you for the suggestion.  Research questions have answered valid academic topics or problems based on knowledge in the field. |
| 2 | Is the protocol technically sound and planned in a manner that will lead to a meaningful outcome and allow testing the stated hypotheses?  The manuscript should describe the methods in sufficient detail to prevent undisclosed flexibility in the experimental procedure or analysis pipeline, including sufficient outcome-neutral conditions (e.g. necessary controls, absence of floor or ceiling effects) to test the proposed hypotheses and a statistical power analysis where applicable. As there may be aspects of the methodology and analysis which can only be refined once the work is undertaken, authors should outline potential assumptions and explicitly describe what aspects of the proposed analyses, if any, are exploratory.  Reviewer #1: Partly  Reviewer #2: Yes | Thank you for the suggestion.  We have added a more detailed explanation on the page 13-14, line 308-327.  Hypothesis testing will be conducted in stages : univariate analysis, bivariate and multivariate analyses. First, the data normality test will be done using Kolmogorov Smirnov tests.  The formula for hypothesis testing will be carried out according to whether the data distribution is normal or not. Size effect of the intervention will be measured using Cohen's formula with the category of strength effectiveness according to Cohen is 0.2 if the effectiveness is less, 0.5 if the effectiveness is moderate and 0.8 if the effectiveness is strong. |
| 3 | Is the methodology feasible and described in sufficient detail to allow the work to be replicable?  Descriptions of methods and materials in the protocol should be reported in sufficient detail for another researcher to reproduce all experiments and analyses. The protocol should describe the appropriate controls, sample size calculations, and replication needed to ensure that the data are robust and reproducible.  Reviewer #1: No  Reviewer #2: No | Thank you for the suggestion.  We have added a more detailed explanation on the page 9-10, line 217-226 and page 9, line 209-210.  In the previous manuscript, the calculations were still not precise, so the calculations were changed manually to ensure accuracy.  We have calculated the sample size that will be used with the Lemeshow formula, we found 35 participants. To anticipate the possibility of participants dropping out, a number of samples will be added to keep them fulfilled by the formula : n’ = n/(1-f) so the sample to be used is 39 participants for each group (intervention and control group).  Participants in phase 3, are Patiens with T2DM, family caregivers, and nurses according to the inclusion criteria. The experiment and control group are determined by the person in charge of PROLANIS at PHC based on geographic location and PROLANIS activity evaluation data. |
| 4 | Have the authors described where all data underlying the findings will be made available when the study is complete?  The PLOS Data policy requires authors to make all data underlying the findings described in their manuscript fully available without restriction, with rare exception, at the time of publication. The data should be provided as part of the manuscript or its supporting information, or deposited to a public repository. For example, in addition to summary statistics, the data points behind means, medians and variance measures should be available. If there are restrictions on publicly sharing data—e.g. participant privacy or use of data from a third party—those must be specified.  Reviewer #1: No  Reviewer #2: No | Thank you for the suggestion.  This study will provide opportunities for other researchers who need the same data by contacting the corresponding author. Dissemination of findings and data sharing will be done after this research is completed. (page 17, line 387-389). |
| 5 | Is the manuscript presented in an intelligible fashion and written in standard English?  PLOS ONE does not copyedit accepted manuscripts, so the language in submitted articles must be clear, correct, and unambiguous. Any typographical or grammatical errors should be corrected at revision, so please note any specific errors here.  Reviewer #1: No  Reviewer #2: No | Thank you for the suggestion.  Any typographical or grammatical errors have been fixed. We have attached proofreading certificate. |
| 6 | Review Comments to the Author from reviewer #1  The protocol entitled “Effectiveness of Android-based Integrated Diabetes Self-Management (IDSM) Application improving self-management and glycemic control of type 2 diabetes mellitus patients in indonesia: a mixed methods study protocol” expected to help optimize the implementation of diabetes self-management which can improve glycemic control of Patients with T2DMs as one of the indicators of the Chronic Disease Care Program [Program Pengelolaan Penyakit Kronis (PROLANIS)] program in the PHC setting. The authors emphasized that there is no research publication that develop an Android-based integrated diabetes self-management application in Indonesia. However, many studies those application-based diabetes control in different countries of the World are available in the literature. The novelty of this study is not well described. The organizing of the manuscript is little complicated. The information periodically turns to the data analyze. The writing style must move away from a thesis style. I suggest to the authors to reorganize and summarize some parts for better understanding. Also, I believe there is no need to make a data analyze table. | Thank you for the suggestion.  The novelty in this study has been described in detail. (page 5, line 105-109)  Thank you for the suggestion  In order to better understanding. It has been rearranged, summarized and removed the table regarding analysis explanation particularly on page 13-14, line 308-327. |
| 7 | Review Comments to the Author from reviewer #2   1. Use the same format of referencing in whole paper. There are (2) and [3]. | Thank you for the suggestion.  We have made the same format of reference in whole paper (page 3, line 56-60) |
|  | Review Comments to the Author from reviewer #2   2. There is no definition about diabetes self-management. | Thank you for the suggestion.  We have added the definition of diabetes self- management in page 3, line 69-71 and added references. |
|  | Review Comments to the Author from reviewer #2   3. As you have a separate section called “study aims”, it is better to remove the last two line of “Background” that you wrote the aim of the study | Thank you for the suggestion.  We have removed the last 2 lines in the background about the objectives of the research in page 5, line 108-109. |
|  | Review Comments to the Author from reviewer #2   4. Will all the study phases be conducted in Public Health Center (PHC) in Yogyakarta city? How about for family members? How you will reach them? | Thank you for the suggestion.  We've added an explanation of how to reach families in page 7, line 160-161    In Indonesia, family involvement in health care is very important, so that patients who will check their health at PHC are often accompanied by their families. This makes it easier for researchers to reach families. However, they are still being followed up by phone call and visiting to their house. |
|  | Review Comments to the Author from reviewer #2   5. What are the reason to do FGDs among patients in Phase 1? Will it let patients to participate in discussion by considering their own unique experiences? How about their gender, religious …composition? | Thank you for the suggestion.  We have added an explanation about the reason to do among patients in phase 1 in page 7, line 164-166.  Gender, religion, and socioeconomic status are highly considered when we form the FGD groups to ensure that participants will feel comfortable expressing their unique experiences during the FGD process, so that researchers will get lots and varied information from the FGDs conducted. |
|  | Review Comments to the Author from reviewer #2   6. Will other types of diabetes like T1DM… be considered as exclusion criteria? | Thank you for the suggestion.  We have added T1DM patients to the exclusion criteria in page 11, line 254. |
|  | Review Comments to the Author from reviewer #2   7. How about a family member who has T2DM? will he/she be excluded? | Thank you for the suggestion.  We have added family with DM as exclusion criteria on page 11, line 258. |
|  | Review Comments to the Author from reviewer #2   8. What is sampling method for doctors? | Thank you for the suggestion.  Sampling for physicians will use a purposive sampling technique in accordance with the criteria in page 11, line 261-263. |
|  | Review Comments to the Author from reviewer #2   9. Except “Peer debriefing and member checking” what other things you will do to increase trustworthiness? What about credibility? Transferability,.. | Thank you for the suggestion.  We have added an explanation of credibility and transferability in page 8, line 179-182 with the following sentence :  “We will ensure credibility by doing triangulation of methods (conducting FGD and interviews) and data sources (involving data from patients, families, nurses and physicians. To ensure transferability in this study we will later provide detail, clear, and systematic description of the research results.” |
|  | Review Comments to the Author from reviewer #2   10. How and why you will choose “two PHCs” for data collection in phase 3? Why not more? | Thank you for the suggestion.  The number of sample needed in this study is 78 (39 participants for the control group and 39 participants for the experiment group). This number of respondent will be available at 2 PHCs. In case we get lower number, we will expand respondent recruitment to other PHCs until we reach 78 respondents. (page 9, line 217-223 & page 9, line 211-213. |
|  | Review Comments to the Author from reviewer #2   11. There is no any information about data collection tool for qualitative phases? How they will be developed and used? How about the translation? How long interviews will be conducted approximately? Where the interviews/FGDs will be conducted? | Thank you for the suggestion.  We have addded information about data collection tool for qualitative phases.  FGDs will be conducted to determine knowledge, abilities, barriers and needs of Patiens with T2DM, families and health care providers in carrying out diabetes self-management referring to the pillars of DM management (page 7, line 157-159).  The FGD will be held for approximately 60 minutes online using zoom video conference platform. It will be guided by researcher and assisted by two nurses with master degree qualification who act as note taker and observer (page 7, line 161-163).  The interview will be conducted by the researcher for 45-60 minutes in a closed room at PHC. Participant recruitment is determined by the person in charge of PROLANIS from the PHC (page 7, line 170-172).  The FGD and interview guidelines were made based on references to self-management and the pillars of DM management (page 7-8, line 174-176). |
|  | Review Comments to the Author from reviewer #2   12. There is no more information about study procedure. How the target groups for different phases will be chosen? Who will be involved to recruit or conduct interviews? | Thank you for the suggestion.  We have added information about the study procedure especially the selection of target groups at different phases.  In phase 1, Participants will be divided into three groups, patients with T2DM, families and health care providers group. Ten PHCs will be randomly selected and participants will be selected by the person in charge in PROLANIS at each PHC according to the inclusion criteria. (page 7, line 154-157).  Participants in phase 2 are Patiens with T2DM, families and health care providers who were involved in the phase 1. Five participants in each group will be randomly selected (page 8 line 196-197).  Participants in phase 3 are Participants in this phase are Patiens with T2DM, family caregivers, and health care providers according to the inclusion criteria. The experiment and control group are determined by the person in charge of PROLANIS at PHC based on geographic location and PROLANIS activity evaluation data (page 9, line 208-211). |
|  | Review Comments to the Author from reviewer #2   13. How about **ethical considerations** like using information sheet? Consent form? How many versions you use? How about those who can’t read or write? | Thank you for the suggestion.  Participants who agree and willing are asked to sign the consent form with witnesses as proof of willingness. For participants who cannot read or write, an explanation about the research and informed consent will be read for them by researcher and then participants are asked to give a thumbprint as proof of their willingness to take part in the study (page 15, line 354-356).  We also attached Ethical approval from the Medical and Health Research Ethics Committee of the Faculty of Medicine, Public Health and Nursing Universitas Gadjah Mada with the number of: KE/FK/1068/EC/2021 on September 27, 2021 (page 15, line 359-361) |
|  | Review Comments to the Author from reviewer #2   14. Please use reference to support “Measurement of self-management scores will be done three times each month after the intervention..” | Thank you for the suggestion.  We have added a reference to support measurement of self-management scores will be done once a month for three months after the intervention in page 10, line 236-239.  In behavior change theory, Based on Lally & Gardner (2013), it takes 21 days to form a habit in changing behavior. So that changes in behavior can be measured every month. |
|  | Review Comments to the Author from reviewer #2   15. One of the questions is that considering only Patients with T2DMs who use Smartphone and can operate it will make ethical issues? What about those patients who can’t buy or will not have smartphone? | Thank you for the suggestion.  Patients with T2DM who do not have a smartphone will be excluded, because in Indonesia, based on a preliminary study, most of them already have a smartphone. Patients who can’t buy or will not have smartphone will be given a module after research finish (page 15, line 356-357). |
|  | Review Comments to the Author from reviewer #2   16. This paper should be edited by an English native. For example, **“Phase 2, phase 2b will use the Think Aloud (TA) method. In this phase the researcher tested the Android-based IDSM Application to the user t to find problems in operating it. Participants who will involved are participants who have been given an explanation and give a consent.”** | Thank you for the suggestion.  This paper has been edited by the Senior Consultant of Academic Writing and English Literacy of Faculty of Medicine, Public Health, and Nursing, Universitas Gadjah Mada. |
